# Discovering latent node Information by graph attention network

**DOI:** 10.1038/s41598-021-85826-x

**Published:** 2021-03-26

**Authors:** Weiwei Gu, Fei Gao, Xiaodan Lou, Jiang Zhang

**Affiliations:** 1 Information Science and Technology, Beijing University of Chemical Technology, Beijing, 100029 People’s Republic of China; 2 School of Systems Science, Beijing Normal University, Beijing, 100875 People’s Republic of China

**Keywords:** Scientific data, Information technology, Applied physics

## Abstract

In this paper, we propose graph attention based network representation (GANR) which utilizes the graph attention architecture and takes graph structure as the supervised learning information. Compared with node classification based representations, GANR can be used to learn representation for any given graph. GANR is not only capable of learning high quality node representations that achieve a competitive performance on link prediction, network visualization and node classification but it can also extract meaningful attention weights that can be applied in node centrality measuring task. GANR can identify the leading venture capital investors, discover highly cited papers and find the most influential nodes in Susceptible Infected Recovered Model. We conclude that link structures in graphs are not limited on predicting linkage itself, it is capable of revealing latent node information in an unsupervised way once a appropriate learning algorithm, like GANR, is provided.

## Introduction

We are surrounded by various relations, in which, rich information is behind. For example, nodes that bridge different communities play a vital role in information sharing^1^; who you coauthor with in scientific research is highly related to the success of your research career^2,3^; some unique niche of species may become the bottleneck of the energy flows in the entire food web chain^4^; the linked web pages rather than the contents that play more vital roles in ranking web pages^5^. Thus unraveling the latent information and values of nodes is of great significance in both theory and application. It is one of the most important tasks in network analysis.

A bunch of node centrality measures have been proposed to uncover nodes’ importance. For example, degree, clustering coefficient, betweenness, average distance, all those indicators can be computed directly^6^. There are also some task oriented methods such as PageRank for webpages^5^, node impact factor for trade flow networks^7^, energy bottleneck index^4^ for food webs, and disruption index^8^ for citation networks. All these ingenious indicators are hand-crafted, prior domain knowledge and human heuristics are required. Besides, they are designed only for node centrality measuring task and can hardly be transferred to solve other problems such as node classification and graph visualization.

Network representation learning tackles the above mentioned problems by embedding nodes into a low-dimensional space ${\mathbb {R}}^d$, such that the local and global relational information can be decoded in the dense representation vectors. According to the depth of neural networks, the network representation algorithms can be divided into 2 categories. The first category mainly includes some shallow neural network based algorithms such as DeepWalk^9^, node2vec^10^, LINE^11^, HOPE^12^ and struc2vec^13^, those algorithms decode graph structure into dense vectors with shallow neural network architectures and apply node classification, node ranking, as well as link prediction^10,14^ tasks to evaluate the embedding qualities of algorithms. Although handcraft features can be avoided, special embedding skills, such as different random walk strategies^10^ are inevitable. Also, these shallow neural based embedding approaches can hardly incorporate node attributes, for example the abstracts or the main text of papers in citation networks. Besides, due to the limited adjustable parameters^15,16^ the predictive accuracies of the downstream tasks are low. The second embedding category solves the low accuracy problem via incorporating more adjustable parameters, inspired from deep learning techniques in image processing^17^ and natural language processing^18^, some deep learning based algorithms such as graph convolutional network (GCN)^15^, graph attention network (GAT)^16^, VGAE^19^, ARVGA^20^ and other various graph neural networks^21–25^ have been proposed.

Among those deep learning architectures, GAT computes representation of nodes by combining neighborhoods’ vectors in an adaptive way with a self-attention mechanism^26,27^. The attention weights are adjustable parameters computed by aggregating neighbor vectors, those vectors can be updated dynamically according to the state of nodes within a local connected neighborhood. Attention weights can alter the values according to node pairs’ context during the decision stage instead of the learning phase. GAT algorithm takes node labels as supervised information and can achieves high node classification accuracy. However in real world, node labels are rare. There are only some citation networks and protein interaction networks that have supervised node label information. The scarcity of node labels prevents GAT to be applied in domains such as social network, biological networks and etc.

In this paper, we propose that compared with node labels, supervised linkages are more suitable for extracting meaningful network representations to perform the visualization, node classification, and community detection tasks, since linkages not only contain richer nodes’ popularity^28,29^ but also encode nodes’ similarity information^30^.

Taking linkages as supervised information serves at least two benefits. First, we do not need extra node label information, GAT can expand its application domains from citation network to social network, ecological network, protein interaction network and so on. Second, the training corpus (node pairs) increases with the square approximately of the network size, which is much faster than the linear growth of node labels, in this way the deep models can be trained sufficiently, predicting links is equivalent to reconstruct  the graph^19,31^. Although link prediction based graph representation algorithms have been discussed in classic deep models^15,19,31–33^, the importance of linkages has not been fully stressed since classic graph convolution based models mainly focus on node classification or graph classification tasks. Besides, vital node identification as one of the most important tasks of network analysis, has been ignored in these classic deep learning frameworks.

In this paper, we extract meaningful attention weights and network representations by proposing GANR with a fixed sampling strategy. GANR takes the node pair relations as the supervised information. A variety of experiments show that compared with other graph neural network based representations such as VGAE^19^ and ARVGA^20^, GANR not only acquires a comparative link prediction accuracy but can also obtain high quality node representations and valuable attention weight matrix that can be applied into a variety of downstream tasks. We discover that the nodes with more attention been paid by their neighbors have more vital status in networks. The vital nodes identified by attention weight matrix are elites in a Chinese co-investment network, the most popular papers in the APS citation network or the most influential nodes in disease spreading and information diffusion networks. We also discover that the vector representations extracted from the last layer of the well-trained GANR can achieve higher node classification accuracy compared with other unsupervised node classification algorithms. Its advantage becomes much more significant when the labeled nodes are scarce.

## Results

In order to evaluate the performance of GANR and explore its potential applications, we conduct link prediction, node centrality measuring, node clustering and node classification tasks over several networks, that include Cora (2708 nodes and 5429 edges), Citeseer (3327 nodes and 4732 edges), and APS (1012 nodes and 3336 links). APS is a sub-graph extracted from American Physical Society journals. We also include a venture capital investors (VC) network (1436 nodes and 2265 edges). The venture capital co-invest network is corresponding to the co-invest events from year 1991 to 2005. This network is built on the SiMuTon^34^ database. Cora and Citeseer networks take the abstracts of papers as node attribute features. The dimensions of features are 1433 and 3703 respectively. For APS and VC networks, we use the sparse adjacency vectors of nodes to form raw feature input.

Our results are organized in the following order: first, we represent the link prediction accuracy of GANR over several networks and compare its performance with other competitive link prediction algorithms; second, we show that the well-trained nodal attention matrix of GANR is capable of identifying vital nodes compared with other well-known node centrality measuring algorithms; third, we extract node representations from the well-trained GANR architecture and evaluate its quality by node clustering and node classification tasks, the results show that GANR has a competitive performance compared with other well-known graph representation learning algorithms.

### Link prediction

GANR takes the connectivity of node pairs as the supervised learning information. The goal of GANR is to predict the connection probability between node pairs. To generate GANR’s training corpus, as described in the section ’Training GANR’, we first use the node pair relations in the training set to train GANR, we then evaluate GANR’s link prediction accuracy with the node pairs’ connectivity in the test set.

We select several well-known link prediction algorithms to compare with, that includes classic node similarity based algorithms such as resource allocation (RA)^35^. RA is a traditional link prediction method, and the similarity between two nodes is measured by computing neighbors’ weights which are negatively proportional to its degree. LINE^11^ minimizes a loss function to learn embedding while preserving the first and the second-order neighbors proximity among vertices in the graph and node2vec^10^ adopts a biased random walk strategy and applies Skip-Gram to learn vertex embedding. This embedding algorithm is widely used in recent years. We also compare GANR with some graph convolutional based algorithms such as GraphSAGE^32^, VGAE^19^ and ARVGA^20^. GraphSAGE learns node embedding through a general inductive framework consisting with several feature aggregators. It usually adopts supervised node classification task as the evaluation benchmark with the assumption that better embedding algorithm leads to higher node classification accuracy. GAT is the architecture that our model mainly based, it computes representation of each node by combining its neighborhoods vectors in an adaptive way with adjustable attention weights for different neighborhoods. ARVGA uses a variational graph autoencoder to learn embedding and perform the link prediction as the supervised task.

Two standard metrics, Accuracy and AUC (the area under a receiver operating characteristic curve) are used to quantify the link prediction accuracy. As shown in Table 1, GANR outperforms the baseline methods in link prediction accuracy on Cora, Citeseer and APS graphs. GANR also achieves a comparative link prediction accuracy in VC network.

**Table 1:** The comparison of different algorithms over link prediction task under Accuracy and AUC metric.

Accuracy/AUC	Cora	Citeseer	VC network	APS network
GANR (proposed)	**0.87/0.93**	**0.85**/0.91	0.80/0.90	**0.84/0.94**
RA	0.41/0.75	0.32/0.73	0.33/0.76	0.35/0.78
LINE	0.69/0.76	0.67/0.73	0.78/0.84	0.68/0.74
node2vec	0.82/0.92	0.85/0.89	0.77/0.87	0.83/0.89
GraphSAGE-mean	0.83/0.89	0.84/0.90	**0.81**/0.90	0.82/0.88
VGAE	0.75/0.91	0.75/0.90	0.76/0.88	0.76/**0.94**
ARVGA	0.73/**0.93**	0.73/**0.94**	0.72/**0.91**	0.72/0.92

Bolded numbers are best results among the comparisons in the same column.

### Attention centrality

In this part we show that the attention coefficients extracted from the well-trained GANR can reveal hidden node relations and quantify the importance of nodes. Based on the graph structure, GANR can automatically learn directed and normalized attention weights for any connected node pairs through the attention mechanism. GANR is also able to transfer the undirected and un-weighted networks to directed and weighted ones. The direction and attention weights represent the relative nodal importance from the source node to the sink node. Intuitively, the more attention a node attracts, the more influential and vital it is.

In this paper, we measure the influence of nodes by adding its neighborhoods’ attention values towards the central node across the second layer’s heads (see Methods for detail). The attention accumulating process is described in Eq. (1).

1 $$\begin{aligned} \bar{c}_{i} = {\sum _{k=1}^{K_2}\sum _{j \in {\mathcal {N}}_i} \alpha _{ji}^k } \end{aligned}$$

In Eq. (1), $\alpha _{ji}^k$ is the normalized attention coefficient representing the attention amount being paid by node *j* to node *i* from the *kth* attention head, and $K_2$ is the total number of the attention heads from the second layer in GANR architecture. We then rank all nodes according to their attention centrality and name this nodal ranking algorithm as Attention Rank. We find that the Attention Rank algorithm is not only able to find the most important nodes in VC and APS networks, but can also identify the most influential nodes during the disease spreading process.

One of the most important questions in venture capital analysis is to identify the leading investors (leading VC) among a large amount of invest activities. Syndication in the Chinese venture capital market is typically lead by some vital VCs which are called “elites”. Those VCs always find good investment opportunities, set up investment plans, and organize invest partners. They usually play a major role during the investment activities, therefore, it is important to identify the vital players in VC network. In this paper we apply Attention Rank on the VC network and compare our ranking order with the top 42 leading VCs (’elite’) identified by questionnaire survey with the Delphi method described in our previous work^36^.

According to the Attention Rank algorithm, compared with the follower players in VC industry, the ’elites’ (leading VCs) always attract much attention. Following a paper published in the Science journal^37^, we sort nodes according to their attention centrality in a decreasing order, we find that 30 out of the top 42 elites are elites in our ground truth list. In Table 2 we listed the the top 16 VCs identified by the Attention Rank and find that all of them belong to the elites of our ground truth set. Besides, we find that the top 16 VCs have a large overlap with a recent released VC ranking list which discussed about the ’best’ venture capitals in China^38^.

**Table 2:** The top 16 VC firms that attract the most attention in Attention Rank algorithm.

Rank	VC name	is_elite	Rank	VC name	is_elite
1	MORE/Shenzhen Capital Group	Yes	9	JAFCO ASIA	Yes
2	IDG Capital	Yes	10	FOETURE Capital	Yes
3	Sequoia	Yes	11	GGV Capital	Yes
4	Legend Captital	Yes	12	Walden International	Yes
5	Goldman Sachs	Yes	13	SBCVC	Yes
6	Intel Capital	Yes	14	DFJ Venture Capital	Yes
7	Northern Light Venture Capital	Yes	15	Qiming	Yes
8	DT Capital	Yes	16	Cowin	Yes

To accurately compare the ranking outcomes with other centrality measuring algorithms, we follow the method used in Webspam competition^39^ and apply the Accuracy which defines as the ratio of the hits on the ground truth to evaluate the performance of different node ranking methods. The ranking results are listed in Table 3.

We also use Attention Rank to find the most popular papers in APS graphs. We follow^40^ to evaluate a paper’s importance by counting the number of citations it received within the first 10 years (c_10) after its publication, and (c_10) is used as the comparison metric. We report the Spearman’s rank correlation coefficient between the Attention Rank and the citation (c_10) counting ranking to evaluate the ranking performance^40^.

We choose several unsupervised graph ranking algorithms to compare with. The first is PageRank^5^, PageRank is widely used in ranking web pages in searching engines such as Google and Baidu. The second is Closeness Centrality and the third is Betweenness Centrality^41^. The Closeness Centrality believes the vital nodes should have shorter path lengths to other nodes, while the Betweenness Centrality assumes that the most important nodes should be involved in more shortest paths. From Table 3 we find that the ranking results of GANR significantly outperforms other node centrality ranking algorithms.

**Table 3:** Ranking performance comparison between different unsupervised ranking algorithms.

Dataset	Evaluation	PageRank	Closeness	Betweenness	Attention centrality
VC	Accuracy	0.65	0.60	0.58	0.72
APS	Rank coor.	0.32	0.08	0.03	0.42

To further validate the effectiveness of the Attention Rank algorithm, we apply SIR (Susceptible Infected Recovered) model to examine the spreading influence of the top ranked nodes by various centrality measures^42–44^. SIR model is used for simulating the spreading of virus or ideas on networks. According to the SIR model, nodes have three states: Susceptible, Infected, and Recovered. At each step, the infected nodes, randomly pick susceptible neighbors with a probability *p* to infect(for simplicity, we set $p=1$), after that these nodes may recover with the probability 1/*k*, where *k* is the degree of the focal node. Thus, an infected node will infect *k* neighbors at last. The spreading process will stop if there is no infected nodes.

**Figure 1:**
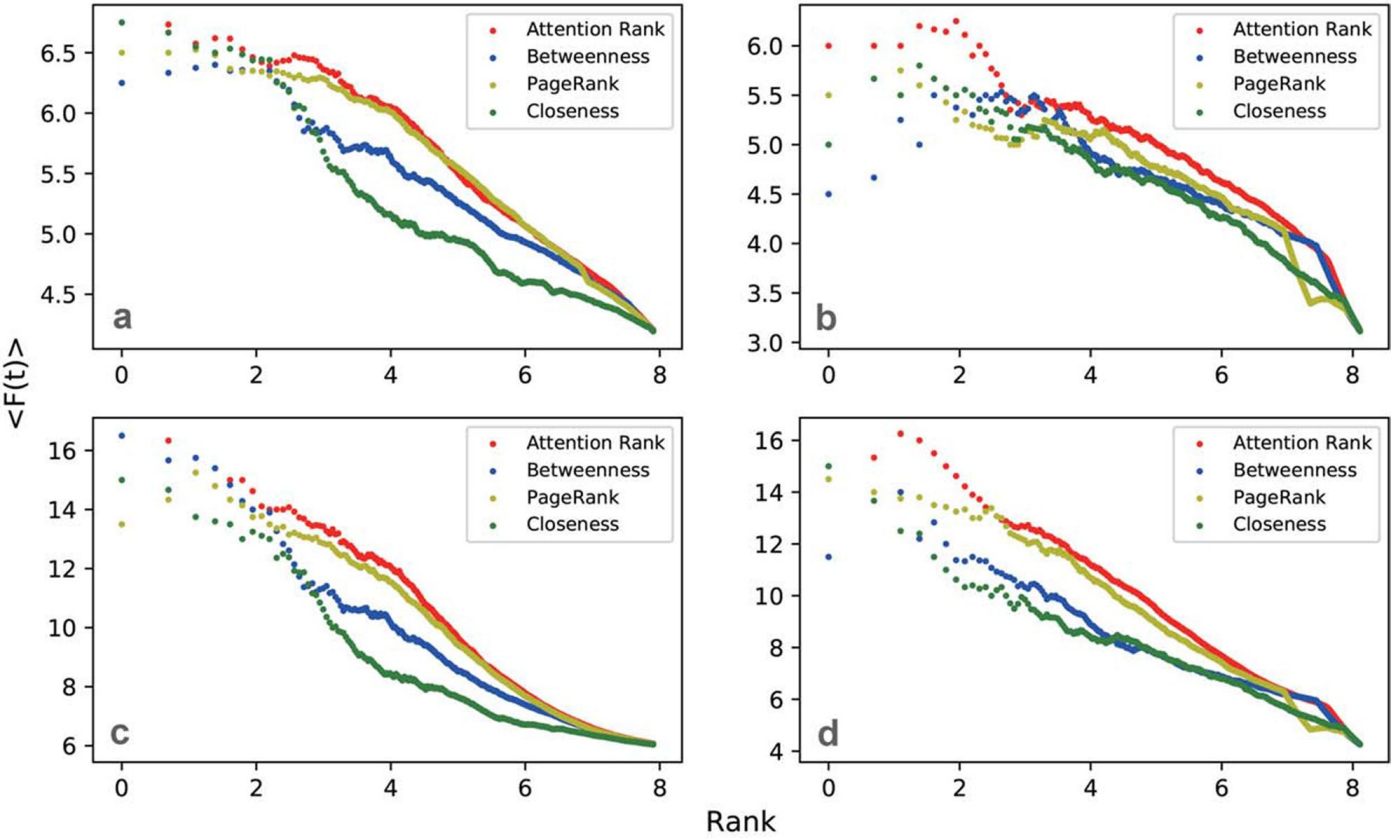
The comparison between the spreading power of different ranking algorithms over $\langle F(t)\rangle$. In panels (**a**) and (**b**), we plot the average number of $\langle F(t)\rangle$ for $t=3$ of the top-L users as ranked by the five centrality measures of Cora (**a**) and Citeseer (**b**) graphs, while in panels (**b**) and (**d**), we plot the average number of $\langle F(t)\rangle$ for $t=9$ of Cora (**c**) and Citeseer (**d**) graphs.

To evaluate the influence of the most influential nodes, we set nodes within rank *k* as the spreading seeds and implement the SIR infectious process. The total number of infected and recovered nodes at time *t*, denoted as *F*(*t*) which is an indicator to evaluate the influence of the seed nodes. Clearly, *F*(*t*) converges to a stable number $F(t_c)$, where $t_c$ is the time when no nodes get infected. The spreading process always takes long time to converge. Thus instead of considering the stable state, we focus on the influence within a shorter time ($t=3$ or $t=9$ in our experiments), since the spreading of the early stages are usually more important in practice. We use $F(t=3,9)$ to measure the spreading influence of the selected seed node during timestamp 3 and 9. We repeat spreding process for 10 times for each node to get the mean value $\langle F(t)\rangle$ over all nodes. We plot the average *F*(*t*) with their ranks by different centrality measures of each node in Fig. 1.

As shown in Fig. 1, the spreading ability of Attention Rank is above other ranking algorithms which represent that the top nodes selected by the Attention Rank are more influential, and this advantage becomes much more significant for larger networks and longer time periods. In summary, the amount of attention that a node gets is a good indicator to measure node’s centrality. We stress that the attention weight matrix extracted from graph is an endogenous network property, hidden in the structure linkage. GANR reveals this property by imposing the attention weights correctly distributed for predicting the linkage existence. Therefore, GANR can fully utilize the information hidden in structure and reveal node attributes.

Compared with other classic node ranking methods, Attention Rank can incorporate node attributes and structural information, and the ranking results can reflect more valuable information and open a new way to rank graph nodes based on deep learning architecture.

### Graph visualization

**Figure 2:**
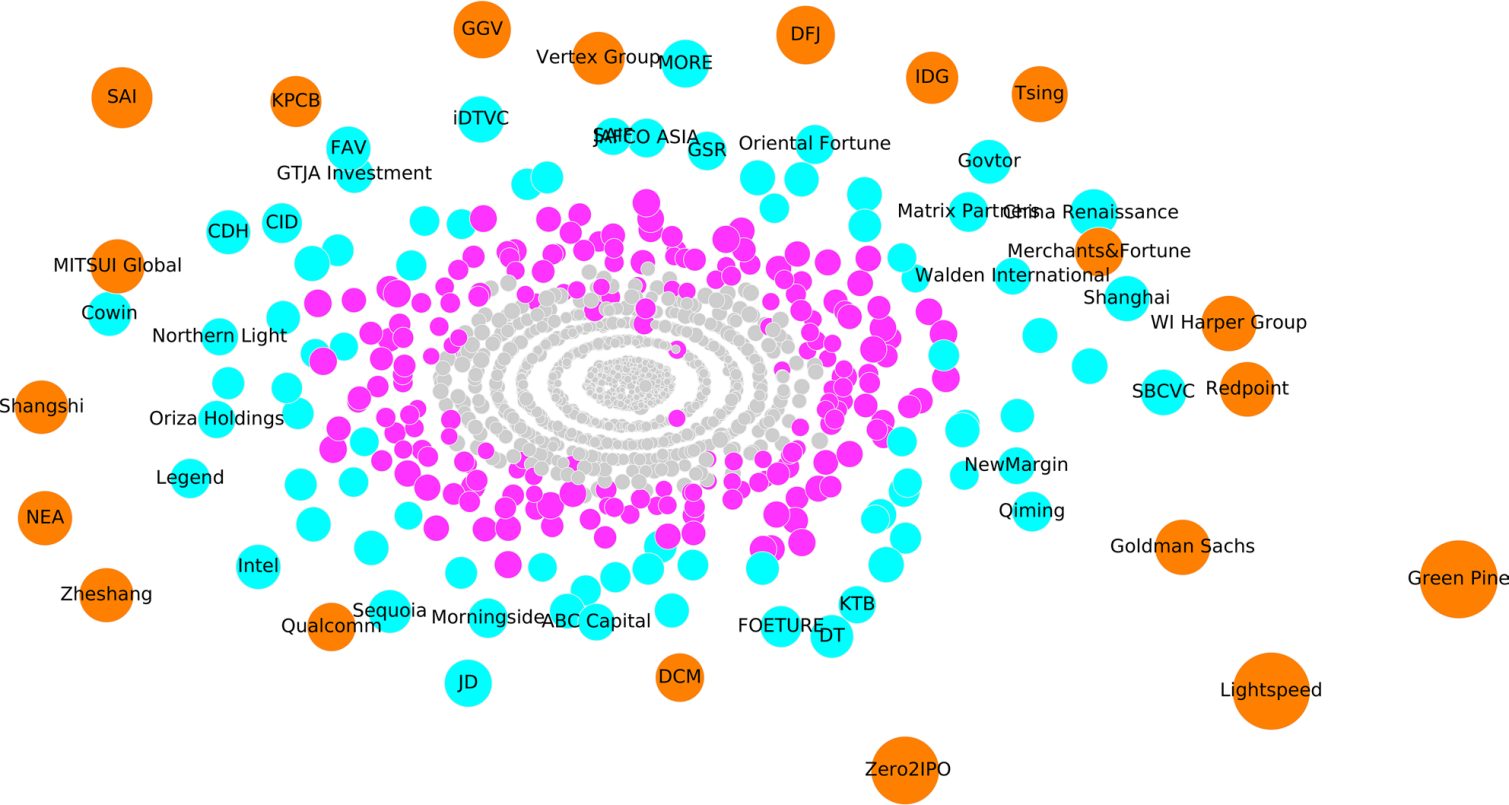
The visualization of the attention matrix extracted from the well trained GANR.

GANR can not only be applied in predicting missing links and measuring nodes centralities but can also provide graphical representation to gain a insightful view of graph’s structure. We first use Multidimensional Scaling^45^ to embed the attention matrix extracted from the well-trained GANR into 2 dimensions. We also divide VCs into 4 categories based on their total attention weights, with the orange color indicate the most important VCs that attract the most attention and the grey color represent the least important nodes that attract the minimum attention. Node size is proportional to the attention weight. From Fig. 2 we find that the ’elite’ VCs such as ’IDG’ and ’Goldman Sachs’ are on the periphery while the most obscure VCs are located in the center. The middle of Fig. 2 is like a black hole, the more cental a node is, the less likely it will become an elite and achieve a higher IPO (Initial Price Offering) rate. The visualization of attention weights can help us evaluate VC’s status in the investing ecosystems.

**Figure 3:**
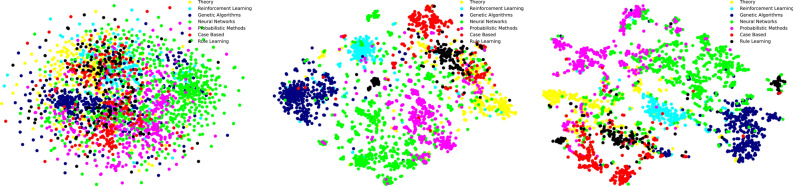
t-SNE visualization of Cora graph from the raw features (left), GraphSAGE-mean (middle), and the GANR (right).

We use t-SNE^46^ to visualize the raw feature input, node representations learned from the second layer of pre-trained GraphSAGE-mean and GANR. Nodes in Fig. 3 represent papers in the Cora citation network with colors denoting papers’ class. The graph representation extracted from GANR is superior to GraphSAGE-mean and raw attributes under the NMI (Normalized Mutual Information) and the Silhouette score metrics. The clusters of the GANR’s representations are clearly defined with a Silhouette score equals to 0.17 compared with 0.06 in GraphSAGE-mean and 0.00 in the raw features. The NMI value of GANR is 0.44 compared with 0.42 in GraphSAGE-mean vectors and 0.13 in the raw features. We find that in general nodes belonging to the same class block together, the community structures of the Reinforcement Learning and the Genetic Algorithms are more significant with the highest sub-network density 0.017 and 0.009 compared with the whole network’s density 0.001. The visualization of GANR can identify graph’s community structures clearly.

### Node classification

Compared with node label information, network representations based on structure learning contain richer information and can be easily collected. In fact, there are a small number of citation networks and protein interacting networks that have supervised node label information. To evaluate the network representation quality of GANR, we compare it with the unsupervised GraphSAGE-mean and the widely used *node*2*vec* on node classification task.

Following the default parameter setting, we extract node representations and feed them into a simple logistic classifier to predict the classification accuracy of the test set. In order to control changing variables, we fix the embedding dimension to 128, and name the embeddings as GANR_128, GraphSAGE-mean_128 and node2vec_128. We first train the logistic classifier with the fixed node representation vectors and take nodes’ label as supervised information. We then test and evaluate the node classification accuracy on the validation set. Finally, we report the average Micro-F1 value for the 10-fold cross validation.

**Figure 4:**
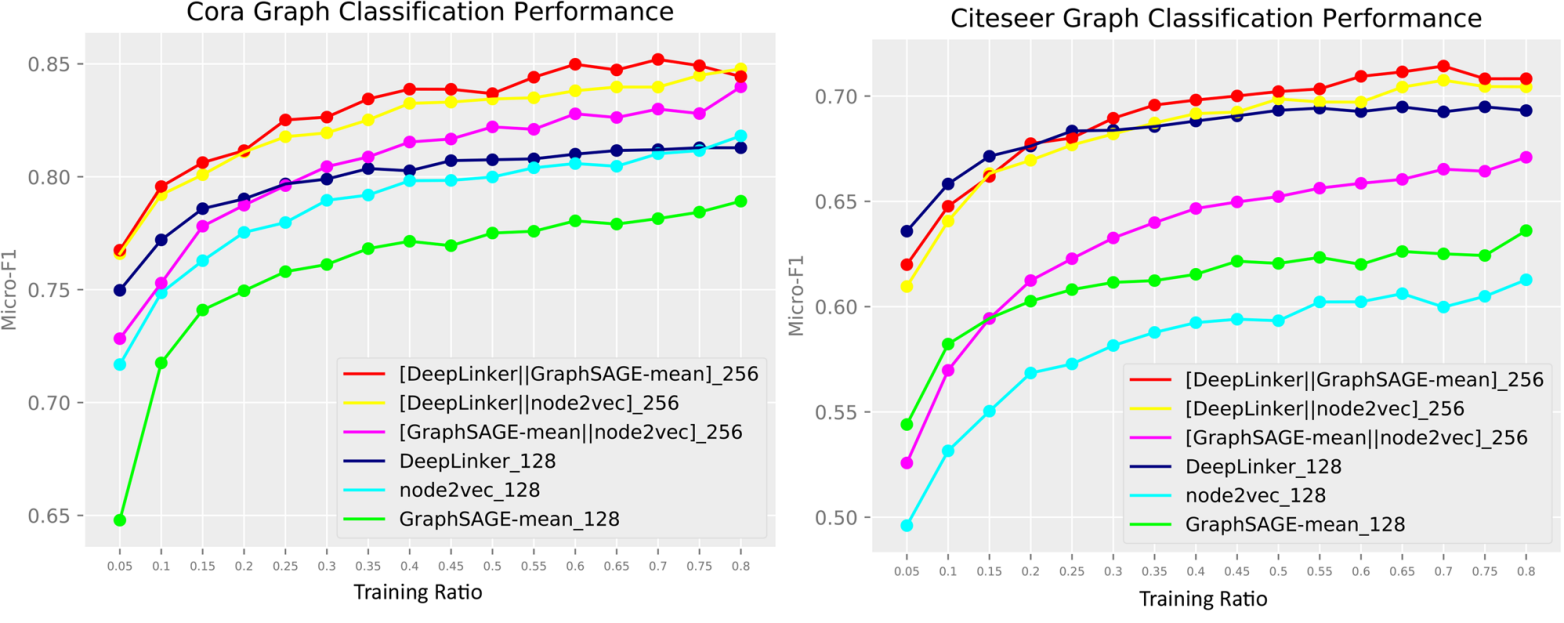
Classification accuracy comparison under different representation learning methods over a variety of training ratio.

The left subfigure of Fig. 4 shows that in Citeseer network, GANR outperforms *node*2*vec* and GraphSAGE-mean especially when the training set is small. In the right subfigure, when the training ratio is less than 10%. GANR also outperforms *node*2*vec* and GraphSAGE-mean. This advantage is important since in real networks there are only few labeled graphs, and manually labelling nodes not only cost a large amount of time but also introduce biases.

Moreover, in order to improve the node classification accuracy, we increase the embedding dimensions by concatenating the vectors learned from different algorithms. As shown in Fig. 4, the concatenation of GANR and GraphSAGE-mean achieves the highest node classification accuracy in Cora and Citeseer compared with other combinations, the combination of GANR and *node*2*vec* has better classification performance compared with the concatenation of GraphSAGE-mean and *node*2*vec*. We notice that the concatenating of GraphSAGE-mean and node2vec performs even worse than GANR in 128 dimension in the Citeseer network. GANR can outperform other methods especially under the scenario when the supervised information is lack or absence.

## Discussion

In this paper, we propose GANR, which is a link prediction based graph representation learning algorithm with a graph attention architecture and a sub-graph sampling strategy. GANR is not only able to predict missing links but can also reveal hidden graph information such as meaningful vertex representation and attention weights. GANR is capable of identifying vital investors, highly cited papers as well as the influential nodes during the virus or information spreading process via node ranking based on the attention centrality. The learned node representations can also used to classify nodes into different groups with a relatively high accuracy especially when the number of labeled nodes are scarce.

Node representations extracted from graph structure learning such as GANR have many advantages than those obtained from node classification based representations since graph structures contain richer information, similar to the language model in natural language processing^27^, link prediction can be used to generate node representations for many downstream tasks, such as node classification, graph classification, visualization and etc.

Despite the tedious process of adjusting the hyper-parameters, we still believe the link prediction based network representation can lead to both quantitative and qualitative leap in network processing. We believe that much more information is hidden in the linkage data of various networks, and ’let the linkage speak itself’ is the right way to reveal structure information. As a common problem of most deep learning based algorithms, GANR has the explanation problem, uncovering the hidden information behind GANR is an important task in our future work.

## Methods

### GAT architectures

To start with, we review the architecture of GAT model on which our algorithm is mainly based. GAT takes a set of node features as input, **h** = {$\vec {h}_{1}$, $\vec {h}_{2}$, ... , $\vec {h}_{N}$}, in which, $\vec {h}_{i}$ $\in$ ${\mathbb {R}}^F$, and *N* is the number of nodes, *F* is the number of node attributes. We use $\vec {h}_{i}' \in {\mathbb {R}}^{F^{'}}$ to denote GAT’s outputs that contain $F^{'}$ node features. The target of GAT is to obtain sufficient expressive power to transfer the input features into high-level output features. It first applies a learnable linear transformation, parameterized by a weight matrix, **W** $\in$ ${\mathbb {R}}^{F^{'} \times F}$ to each node, it then uses a single-layer feed-forward neural network $\vec {a}\in$ ${\mathbb {R}}^{2F^{'}}$ to compute the attention coefficients between nodes. This computation process is shown in Eq. (2), where $.^T$ represents matrix transposition and $\parallel$ is the concatenation operation. Node *j* is a neighbor of node *i*, and $\alpha _{ij}$ indicates the importance of *j*’s features to *i* among all *i*’s neighbors, represented as ${\mathcal {N}}_{i}$.

2 $$\begin{aligned} \alpha _{ij} = \frac{\exp \left( LeakyReLU \left( \vec {a}^{T} [{\mathbf{W}} \vec {h}_{i} \parallel {\mathbf{W}} \vec {h}_{j}] \right) \right) }{\sum _{{j \in {\mathcal {N}}_{i} }}\exp \left( LeakyReLU \left( \vec {a}^{T}[{\mathbf{W}} \vec {h}_{i} \parallel {\mathbf{W}} \vec {h}_{j}]\right) \right) }. \end{aligned}$$

Once the normalized attention coefficient $\alpha _{ij}$ is obtained, GAT aggregates nodes’ features as a combination of their neighbors, followed by a potentially nonlinear sigmoid function $\sigma$, as is shown in Eq. (3).

3 $$\begin{aligned} \vec {h^\prime _i} = \sigma \left( {\sum _{j \in {\mathcal {N}}_i} \alpha _{i,j} {\mathbf{W}} \vec {h}_{j} } \right) \end{aligned}$$

Finally, GAT employs multi-head attentions to stabilize the learning process. **K** denotes the number of attention heads, $\alpha _{ij}^k$ denotes the *k*th relative attention weights of $j's$ features to *i*. The output features of nodes’ neighbors are either concatenated or averaged to form their final output features, as shown in Eq. (4):4

### GANR architecture

**Figure 5:**
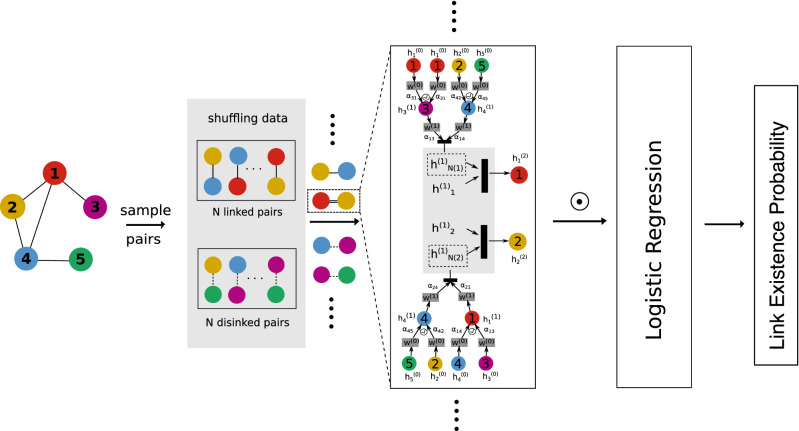
The overall architecture of GANR. We use a simple five-node graph to illustrate GANR’s architecture. We describe the linked relations in solid lines and the unlinked ones in dashed lines. We take the linked node 1 in red and node 2 in yellow as a training example. To start with, we sample nodes 3 and 4 as 1 and 2’s 1st-order neighbors, we then sample nodes 1, 2, 5 as 1 and 2’s 2nd-order neighbors, nodes 1, 2, 5 are also the 1st-order neighbors of nodes 3 and 4. After that we calculate nodes 1 and 2’s vector representations based on the initial features via GAT architecture. We acquire edge vector via calculating the Hadamard product of the 1 and 2’s. Finally a logistic regression function is applied on the edge vectors to compute the linkage existence probability.

The overall architecture of GANR is shown in Fig. 5. An encoder encodes nodes to vector representations, $F^{'}$ $\in$ ${\mathbb {R}}^{F^{'}}$, a decoder then generates edge vectors by aggregating the vector representation of nodes. Finally, a score function is applied to evaluate the link existence probability between two nodes via the edge vectors. One of the key ideas behind GANR is to learn how to aggregate nodes’ features into edge vectors for link prediction task.

As mentioned above, memory bottleneck is the biggest problem which preventing GAT to be applied on large scale networks. Due to the scale-free property of most networks, once hub nodes are sampled as the 1st-order neighbors, then the 2nd-order neighbors can quickly fill up the memory, this property prevents GAT to be applied on larger networks. Besides, the existing GPU-enabled tensor manipulation frameworks are only able to parallelize on the normalized activation coefficients ($\alpha _{ij}$) for the same sized neighborhoods, it prevents GAT from parallel computing.

### Fixed-sized neighborhood sampling

Here we use the fixed-sized neighborhood sampling strategy to solve the memory bottleneck. The undirected graph can be represented as $G=(V,E)$, with *V* denoting the set of nodes and *E* representing edges. As illustrated in Fig. 5, for a given node pair we first use the Hadamard product to form the edge vector and then evaluate the edge existence probability via feeding the edge vector into a logistic regression training model. In GANR we sample a fixed first and second order nodes to form nodes’ neighbors . Taking node *i* as an example, we uniformly sample a fixed-sized set of its neighbors defined as ${\mathcal {N}}_{i}$ from set $\left\{ i \in V: (i,j) \in E \right\}$.

Different from sampling neighborhood during each training iteration in GraphSAGE, we sample only once and keep the neighborhoods fixed during the whole training process that is the major sampling difference between GraphSAGE and GANR. As illustrated in Fig.5, the link prediction results of GANR show that the fixed sampling strategy is more robust, less fluctuate and achieves higher accuracy than the flexible sampling strategy. We compute the Hadamard product between two output vectors to form edge vector, as is shown in Eq. (5), note that $e_{ij}$ is a *d*-dimensional vector.

5 $$\begin{aligned} e_{ij} = (\vec {h^\prime _i} \odot \vec {h^\prime _j}) \end{aligned}$$

We then assume that the probability between node *i* and *j* is given by Eq. (6) where $\theta$ is a *d*-dimensional parameter, and $e_{ij}^{T} \theta$ is the dot product between vectors $e_{ij}$ and $\theta$.

6 $$\begin{aligned} p_{ij}(e_{ij};\theta ) = \frac{1}{1+exp(e_{ij}^{T} \theta )} \end{aligned}$$

### Training GANR

The whole framework is trained by minimizing the following objective function:

7 $$\begin{aligned} {\mathscr {L}} = -\frac{1}{\mid \varepsilon \cup \varepsilon ^{-}\mid } \sum _{{ij}\in \varepsilon \cup \varepsilon ^{-}} y_{ij}\log p_{ij} +(1-y_{ij})\log (1-p_{ij}) \end{aligned}$$

where, $y_{ij}$ is the supervised linkage information between *i* and *j*, with 0 indicates not connected node pairs and 1 for connected node pairs. We first randomly divide the full set of edges *E* into three parts, $\varepsilon$ for training, $\phi$ for validating and $\tau$ for testing. And we also sample the equal amount of disconnected node pairs to form the $\varepsilon ^{-}$, $\phi ^{-}$ and $\tau ^{-}$. Note that $\varepsilon ^{-}$ represents the negative training samples, in which each node pair cannot be connected within 2 hops.

In order to evaluate the performance of GANR and explore its potential applications, we conduct link prediction, node centrality measuring as well as node classification tasks over several networks ranging from citation network to venture capital co-invest network. All experiments are performed under the PyTorch machine learning environment with a CUDA backend.

### Experiments setup

We utilize the following five networks in our experiments:

- Cora network contains 2708 nodes 5429 edges and 7 classes. Each node has 1433 attributes corresponding to elements of a bag-of-words representation of a document.
- Citeseer network contains 3327 nodes 4732 edges, and 6 classes. Each node has 3703 attributes extracted from paper contents.
- APS graph has 1012 nodes and 3336 edges. The adjacency matrix is used as the the one-hot input node attribute in the training process. Follow a paper published on Science^40^ here we quantify papers’ impact and importance by counting the number of citations over 10 years (c_10) after their publication, c_10 is used as ground truth metric for measuring nodes importance.
- VC network is a venture capital co-invest network with nodes representing venture capital companies and edges corresponding to co-invest events. It contains 1436 nodes and 2265 edges. Here we use the adjacency matrix as the one-hot input node attribute in the training process. In this network, 42 venture capitals are identified manually as VC which play a vital role in venture capital events. These ventural capitals are regarded as the ground truth in node ranking task. The VC investment network is built on the SiMuTon^34^ database.

In our experiments, we keep node2vec and LINE’s parameters as they are in the original papers. We set the same parameters for GraphSAGE, GAT and GANR which include the type and sequence of layers, the choice of activation function, placement of dropout, and setting of hyper-parameters.

GANR algorithm consists of two layers, the first layer is made up of 8 ($K1 = 8$) attention heads over all networks. The main purpose of the first layer is to compute the hidden features of the 1st-order neighbors. We then add the non-linearity by feeding the hidden features to an exponential linear unit (ELU), as shown in Eq. (3). The aggregated features from each head are concatenated in this layer.

The main purpose of the second layer is to compute the edge features that used for evaluating link probability. The aggregated features from each head are averaged in this layer. The output of the second layer is the final feature representations for nodes. We then compute the Hadamard distance between two node features to represent the edge vector, as is shown in Eq. (5). Once edge vectors are obtained, an active function sigmoid $\sigma$ is applied to evaluate the edge existing probability between nodes.

We initialize the parameter of GANR with Glorot initialization^47^ and train to minimize the binary cross-entropy, as shown in Eq. (7), for the training set we use the Adam SGD optimizer^48^ with an initial learning rate of $5e-4$. We also apply an early stopping strategy over link prediction accuracy for the validation set with the patience sets to 100 epochs.

We solve the memory bottleneck by sampling a fixed neighbors size (20) for both 1st and 2nd-order neighbors for GANR. The sampling strategy is illustrated in Fig. 5.
